# Effect of Previous Caudal Block to Predict Successful Outcome after Adhesiolysis using a Steerable Catheter in Lumbar Failed Back Surgery Syndrome: A Retrospective Study

**DOI:** 10.7150/ijms.72272

**Published:** 2022-06-06

**Authors:** Ji Yeong Kim, Do-Hyeong Kim, Dong Woo Han, Young Chan Kim, Ji Young Lee, Young Kyung Park, Hue Jung Park

**Affiliations:** 1 Department of Anesthesiology and Pain Medicine, Yonsei University College of Medicine, Gangnam Severance Hospital, Seoul 06273, Republic of Korea.; 2 Department of Anesthesiology and Pain Medicine, Seoul St. Mary's Hospital, College of Medicine, The Catholic University of Korea, Seoul 06591, Republic of Korea.

**Keywords:** Adhesiolysis, Lumbar Failed Back Surgery Syndrome, Post-laminectomy Syndrome

## Abstract

Adhesiolysis is minimally invasive and commonly used for pain associated with adhesion after lumbar spine surgery. Caudal epidural block may be used for radiating pain due to failed back surgery syndrome. We evaluated the predictive value of response to caudal block performed prior to adhesiolysis in failed back surgery syndrome. Between January 1, 2013 and June 30, 2020, 150 patients with failed back surgery syndrome were treated with adhesiolysis using a steerable catheter at the pain clinic of a tertiary hospital after failed conservative treatment (including caudal block). Patient demographics, pain duration, and lumbar magnetic resonance imaging findings were examined. Response to previous caudal block was determined as a binary result (yes or no). Patients were followed up 3 months after adhesiolysis. Successful outcome was defined as a ≥2-point reduction in the numeric rating scale scores for radicular pain 3 months after adhesiolysis, evident in 81/150 (46%) patients. Multivariable logistic regression analysis revealed that caudal block response was an independent predictor of successful adhesiolysis (odds ratio = 4.403; *p* = 0.015). Response to prior caudal block is a positive predictor of successful adhesiolysis.

## Introduction

Failed back surgery syndrome (FBSS) refers to a situation in which the surgical outcome is suboptimal compared with the expectation of the patient and the clinician before the surgery, because the back or radiating pain persists even after the spine surgery 1. The Spine Patients Outcomes Research Trial 2 has reported a reoperation rate of 10%, four years after the initial surgery. Deyo et al. 3 also reported that the possibility of need for repeat surgery after 4 years is 10.6%⎼17.2% in patients who had undergone operations for lumbar spinal stenosis. In addition, the success rate of surgery gradually decreases every time the spine surgery is repeated 4. The overall failure rate of lumbar spine surgery has been consistent at approximately 10-46%, despite the innovations in surgical technologies 5, 6. Consequently, a significant number of patients with FBSS can be expected 7. Further, the activities of daily living and quality of life decrease 1, 8. In addition, because these patients are frequent users of health care services, they have social and economic burdens 5, 9, 10.

The pathophysiology of FBSS is complex 10-15. The possible causes include epidural fibrosis, sacroiliac joint pain, disc herniation, spinal stenosis, arachnoiditis, and facet joint pain, along with inappropriate surgery 16-22. Among them, perineural fibrosis is an inevitable consequence of prior laminectomy 23. However, whether epidural fibrosis is a major cause of pain after lumbar spine surgery is controversial 24-28. Kuslich et al. 29 reported that if there is a scar tissue that causes pain, the nerve root is fixed in one position and becomes vulnerable to tension or pressure. Ross et al. 17 reported that patients with severe epidural fibrosis experience repeated instances of radiating pain compared to patients with limited evidence of scarring. In contrast, Jinkins et al. 30 analyzed a group of 120 patients with recurrent symptoms even after lumbar spine operation and reported epidural fibrosis in 47% patients. Since epidural scar tissue was also present in all asymptomatic patients, fibrosis within the epidural space was deemed less important. Rather, the cause of sciatic pain was attributed to ischemic or toxic changes in the nerve root itself.

Though the contribution of fibrosis in the origin of pain has been debated, caudal epidural injection and adhesiolysis are the most commonly applied interventions among the non-surgical methods for treating chronic pain in FBSS 21, 31-43. The American Society of Interventional Pain Physicians (ASIPP) 44 advocates adhesiolysis in the absence of response to epidural injection. Adhesiolysis is considered an alternative treatment modality when patients with FBSS do not respond to conservative treatment including epidural injection 31, 45, 46.

According to the ASIPP guidelines 44, epidural block for therapeutic purposes can be repeated if the injection shows a 50% or more reduction in pain scores for at least 2 and a half to 3 months, but it should be administered more than 4 times a year. Based on the guideline, we consider adhesiolysis if the duration of pain reduction is less than about 3 months, or in the absence of change in the pain intensity. In this clinical context, we hypothesized that pain reduction after caudal block could be associated with success of subsequent adhesiolysis. To test this, patients who underwent adhesiolysis for FBSS in a tertiary pain center were analyzed. To the best of our knowledge, no reports have analyzed the correlation between the response to caudal block and adhesiolysis. The aim of the study was to determine the association of the response to caudal block, which was performed previously, with the response to adhesiolysis retrospectively using data obtained from medical records.

## Materials and Methods

### Participants

This retrospective study was approved by the institutional review board of the Seoul St. Mary's Hospital, KC20RISI0665 and was conducted in accordance with the ethical principles of the Declaration of Helsinki. This study has been registered on CRIS (Clinical Research Information Service of the Korea National Institute of Health, https://cris.nih.go.kr/cris/index.jsp, KC20RISI0917). The need for obtaining patient consent was waived owing to the retrospective nature of the study and the assessment of only the formal electronic medical records of the patients. We reviewed the medical records of patients diagnosed with FBSS and who had undergone adhesiolysis using a steerable catheter from January 1, 2013 to June 30, 2020, at the pain clinic of a tertiary hospital.

The following were included in the study: (1) patients aged at least 20 years; (2) patients diagnosed with FBSS based on magnetic resonance imaging (MRI) who reported concordant leg and/or back pain; (3) patients with a leg pain duration >3 months; and (4) patients with leg pain severity of ≥4 on an 11-point numerical rating scale (NRS) after receiving conservative treatment, including oral medication and physical therapy. The following were excluded: (1) patients with other possible causes of symptoms (for example, post herpetic neuralgia); (2) patients with malignancy or bleeding tendency; and (3) patients who were lost to follow-up before the third month of the procedure. Finally, 150 patient charts were selected and reviewed.

### First intervention: caudal epidural block

Caudal epidural block was performed in a sterile environment at an outpatient clinic. Ultrasonography was used, and the participants were instructed to lie in the prone position. After sterile preparation, lidocaine was locally infiltrated around the sacral hiatus. Thereafter, the epidural space was accessed with a 22G Tuohy needle, and the exact needle tip position was confirmed by ultrasonography. Then, a mixture of 20 mL of 0.2% lidocaine, 5 mg dexamethasone, and 1500 IU hyaluronidase (Hirax®, 750 IU/mL, BMIKorea, South Korea) was injected through the needle. After the procedure, neurological examination of the patient was performed to confirm the absence of any complication, and the patient was shifted to the recovery room. The patient was recalled for an outpatient visit 2 weeks or a month later, and the doctor inquired whether the caudal block showed any effect, even for a short duration. The patient's answer was obtained from the medical record as a binary result (was the pain reduced compared to the pre-treatment pain? Yes/No). If the effect of reduction in pain scores by more than half using the caudal block could be maintained for more than 3 months, the patient's pain was determined to be effectively controlled by repeating the caudal block. When the analgesic effect for caudal block was insufficient, adhesiolysis was considered as the next step when the pain scores were reduced by less than half or the duration of pain reduction was shorter than 3 months. We divided the cohort group with insufficient response to the caudal block into two groups according to the response degree and duration. If the pain reduction was less than half or more than half but the duration was less than 3 months, patients were considered as 'Yes'. In contrast, patients who did not show any other pain reduction were classified as 'No'. Even in patients who had undergone caudal block several times previously, 'the response to the previous caudal block' was determined based on the response to the caudal block performed closest to the time of the adhesiolysis.

### Second intervention: adhesiolysis using a steerable catheter

Adhesiolysis was performed under fluoroscopic guidance in a sterile operating room with constant monitoring of blood pressure, pulse rate, and oxygen saturation. The patient was instructed to lie in the prone position, and a pillow was placed under the abdomen to minimize the possibility of lumbar lordosis. The fluoroscope was adjusted over the lumbosacral area such that the caudal approach could be used in both the anteroposterior and lateral views. Local infiltration of 1% lidocaine was performed after accurately determining the needle insertion site around the sacral hiatus. A small incision was made at the needle insertion site, and an introducer was inserted into the epidural space through the sacral hiatus using a 15G Tuohy needle. Approximately 3 mL of contrast medium was injected and an epidurogram was obtained to confirm the correct location of the needle in the epidural space. A steerable catheter was inserted through the introducer after the removal of the Tuohy needle under fluoroscopic guidance. One of three types of steerable navigation catheters (Episol®, GSmedical, South Korea; Biovision®, Technologies LLC, United States; and STREED plus®, Seawon Medi-Tech Co., Ltd, South Korea) was used, and the catheter was selected according to the operator's preference. The target level of adhesiolysis was determined in advance by considering the location of the filling defect, patient's symptomatic dermatome, and lumbar MRI findings. When the catheter tip reached the target level, 3 mL of contrast medium was injected to identify the filling defects by examining the flow of the contrast medium into the nerve roots. Adhesiolysis was performed by pushing, pulling, and rotating the catheter near the target area. During this step, the patient may experience pain in the same location as he/she may have experienced preoperatively. After mechanical manipulation, 3 mL of diluted contrast medium was injected to confirm whether satisfactory filling was obtained epidurally and at the targeted nerve root. Then, a mixture of 10 mL of 1% lidocaine, 5 mg dexamethasone, and 1500 IU hyaluronidase (Hirax®, 750 IU/mL, BMIKorea, South Korea) was divided and injected separately into each target. In the event of suspected complications, such as dura mater puncture, the procedure was stopped immediately. Thereafter, neurological examination was performed, and the patient was discharged from the hospital only after repeated normal test results.

### Data collection

We obtained clinical data pertaining to age, sex, body mass index, duration of symptoms in the past month, intensity of radiating leg pain as quantified using the NRS, per oral opioid use, and past medical history. Radiographic and MRI findings were reviewed to evaluate the severity of lumbar spinal stenosis, presence of spondylolisthesis, and past surgery history (e.g., fusion, discectomy). The severity of stenosis was graded according to the standard classification 47, 48. For patients with multilevel central or foraminal stenosis, the level with the greatest stenosis was selected.

### Clinical evaluation

The NRS was used to compare the intensity of leg pain between pre-treatment and 3 months after treatment. The NRS represents no pain as 0 and the worst pain imaginable as 10. Patients were categorized according to their response to adhesiolysis after 3 months as follows: (1) Patients in whom the NRS score decreased by 2 points as compared to the pretreatment score were defined as responders; (2) patients who required increasing dosages of opioids in the follow-up period of 3 months were defined as non-responders; (3) patients who received lumbar radiofrequency ablation or were treated using spinal cord stimulators after neuroplasty were defined as non-responders; and (4) patients referred to the department of surgery were also defined as non-responders. According to these definitions of response, patients were categorized into responders and non-responders, 3 months after the procedure.

### Statistical analysis

Patient characteristics (continuous variables) were compared between the groups using the Student t-test or Mann-Whitney U test. Categorical demographic data were analyzed using the Pearson chi-square test or Fisher's exact test. *p* values less than 0.05 were considered statistically significant. The paired t test was used to compare the NRS pain scores between pre- and post-procedures. Binary logistic regression techniques were used to quantify the relationship between successful outcomes and patients' clinical and demographic characteristics. To determine the independent positive prognostic factors of the procedure, multivariable logistic regression analysis was performed for statistically significant variables determined via univariable analysis using the enter method. All data were analyzed using SPSS version 24.0 (SPSS Inc., Chicago, IL, US).

## Results

We reviewed the medical records of 150 patients who had undergone adhesiolysis from January 1, 2013, to June 30, 2020. The basic demographic data of the 150 patients and the clinical data for non-responders and responders 3 months after adhesiolysis are shown in Table 1. Severe foraminal stenosis was evident in 14 (9.3%) patients. Forty patients (58%) showed response to previous caudal block.

Table 1 shows the comparison of demographic and clinical variables between the responder and non-responder groups. Among the entire cohort, 69 patients were categorized as responders (69/150 = 46%) and 81 patients as non-responders at the 3-month follow-up. Pearson's chi-square test revealed that the grade of foraminal stenosis (*p* = 0.039) and response to previous caudal block (*p* = 0.026) were associated with successful outcome of adhesiolysis. Milder was the degree of foraminal stenosis, greater was the pain reduction evident 3 months after the procedure if a response to previous caudal block was evident. In patients with mild foraminal stenosis, the success rate of adhesiolysis at 3 months was 56.4%: 40.6% in those with moderate foraminal stenosis and 28.6% in those with severe. The grade of central stenosis showed no significant relationship with the effect of the procedure (*p* = 0.428).

Baseline NRS pain scores were not significantly different between the responder and non-responder groups (p = 0.778).

Table 2 shows the factors associated with outcome assessed using both univariable and multivariable analyses. Statistically significant demographic variables according to univariable analyses were spondylolisthesis (p = 0.076), grade of foraminal stenosis (mild versus moderate, p = 0.159; mild versus severe, p = 0.072), and response to previous caudal block (p = 0.028). Among these selected clinical variables, multivariable logistic regression analyses revealed response to previous caudal block (adjusted odds ratio (OR) = 4.403, p = 0.026) to be a positive predictor of successful adhesiolysis in patients with FBSS. Grade of foraminal stenosis was marginally dependent on successful response (mild versus severe, adjusted OR = 0.137, p = 0.052).

No complications were observed during the caudal block. However, 13 cases of suspected dura puncture were evident during adhesiolysis. Three patients complained of temporary discomfort immediately after the procedure but showed improvement within 2 days. No patient required any further treatment. There was no case of severe neurologic complications such as motor weakness.

## Discussion

The results of our study showed that if NRS pain score for radiating pain was reduced by caudal block performed prior to adhesiolysis even for a short-term period, a successful outcome to adhesiolysis after 3 months is more likely. This is meaningful as this is the first report to show an association between the responses to adhesiolysis and caudal block.

The results of this study suggest the possibility of a common mechanism of action for caudal block and adhesiolysis. FBSS can have multiple etiologies. We hypothesized that in patients who had received caudal injection without any response, facet or sacroiliac joint dysfunction could be the main cause of the pain; hence, subsequent adhesiolysis would not have any effect. Although it is clear that caudal block and percutaneous adhesiolysis are different procedures, there are some similarities. The caudal block targets multiple spine levels at the same time to permit the diluted local anesthetic to reach the target site, and adhesiolysis removes the deleterious effects of scar formation, followed by target delivery of a diluted local anesthetic. Additionally, lidocaine prevents sensitization of the sympathetic arc, has anti-inflammatory effects, and blocks the axonal transport of nerve fibers 49-52. However, this study is not about mechanisms or etiology, nor does it provide data that supports such correlation.

FBSS with prominent radicular symptoms was the subject of our study. Axial low back pain in FBSS is partially non-neuropathic pain, whereas radicular symptoms associated with FBSS could be neuropathic 53-55. According to the 2021 ASIPP Comprehensive Evidence-Based Guidelines, caudal injection is moderately to strongly recommended in post-surgery syndrome for long-term improvement 44. To our knowledge, the effectiveness of epidural injection in neuropathic pain associated with FBSS has not been evaluated. However, as epidural injection is effective in radiculopathy, is easy to perform, and the side effects are rare, caudal injection may be attempted before invasive treatment, such as spinal stimulation, in patients showing no response to conservative treatment. Percutaneous adhesiolysis is strongly recommended for long-term improvement after failure of conservative management and fluoroscopy-guided epidural injection 44. The exact mechanism by which adhesiolysis is effective for neuropathic pain associated with FBSS is unclear. Nevertheless, we believe that as adhesiolysis is effective in some patients, it should be attempted before performing the relatively invasive spinal cord stimulation.

Additionally, grade of foraminal stenosis might also be independently associated with successful response 3 months after adhesiolysis. The variable showed a marginal significance (*p* = 0.052), probably owing to the retrospective design of the study.

There are several limitations to our study. First, the follow-up period is short. Therefore, the power of the study may be low. Second, the retrospective study design is associated with greater researcher bias than the prospective design. Third, we did not include a placebo group. However, even if normal saline is injected instead of lidocaine, mechanical adhesiolysis is inevitable. Therefore, a sham group could not be formed owing to the characteristics of the study. Fourth, ultrasonography was used during caudal injection. In the clinic where the study was conducted, caudal injection is commonly performed, but owing to time constraints, it is not performed fluoroscopically. Therefore, we had to perform ultrasound-guided caudal injection. According to the 2021 ASIPP guideline 44, identification of the caudal space by ultrasonography is inappropriate and caudal injection under ultrasonic guidance is an unproven technology and cannot be accurately targeted. However, Park et al. 56 compared ultrasound-guided and fluoroscopic-guided caudal epidural injection in unilateral lumbar radicular pain and reported that pain and function decreased in both groups after 12 weeks.

FBSS is a complex pathophysiological entity that requires a multidisciplinary approach 57-60, but caudal block and adhesiolysis can be considered the treatment options in selected patients. The grade of foraminal stenosis according to lumbar MRI and response to previous caudal block can be helpful in the selection of patients for adhesiolysis while treating persistent pain associated with FBSS.

## Figures and Tables

**Table 1:** Comparison of demographic characteristics between non-responders and responders

	Total patients (*n* = 150)	Non-responders (*n* = 81)	Responders (*n* = 69)	*p* value
Age (years), mean ± SD	66.7 ± 12.6	66.6 ± 13.1	67.9 ± 11.9	0.265
Sex (male), n (%)	63 (58%)	33 (40.7%)	30 (43.5%)	0.735
Body mass index (kg/m^2^), mean ± SD	24.3 ± 3.6	24.1 ± 3.4	24.4 ± 3.9	0.750
Pre-procedural intensity of leg pain (NRS score), mean ± SD	7.0 ± 1.6	7.1 ± 1.6	7.0 ± 1.6	0.778
Duration of pain (months), mean ± SD	72.1 ± 69.6	78.5 ± 69.4	64.5 ± 69.6	0.220
**Number of previous spine surgeries, n (%)**				0.951
1	117 (78%)	67 (82.7%)	50 (72.5%)	
2	23 (15.3%)	5 (6.2%)	18 (26.1%)	
3	10 (6.7%)	9 (11.1%)	1 (1.4%)	
Diabetes, n (%)	38 (25.3%)	20 (24.7%)	18 (26.1%)	0.845
Hypertension, n (%)	71 (47.3%)	37 (45.7%)	34 (49.3%)	0.660
Spondylolisthesis, n (%)	29 (19.3%)	20 (24.7%)	9 (13%)	0.072
**Central stenosis, n (%)**				0.428
Mild	54 (36%)	27 (50.9%)	27 (56.3%)	
Moderate	24 (16%)	12 (22.6%)	12 (25%)	
Severe	23 (15.3%)	14 (26.4%)	9 (18.8%)	
Foraminal stenosis, n (%)				0.039*
Mild	55 (36.7%)	24 (45.3%)	31 (64.6%)	
Moderate	32 (21.3%)	19 (35.8%)	13 (27.1%)	
Severe	14 (9.3%)	10 (18.9%)	4 (8.3%)	
**Response to previous caudal block, n (%)**				0.026*
Yes	40 (58%)	14 (43.8%)	26 (70.3%)	
No	29 (42%)	18 (56.3%)	11 (29.7%)	

*p* values were obtained using Pearson's chi-square test or Fisher's exact test and the Student t-test or Mann-Whitney test; * indicates significant differences; NRS = numeric rating scale; SD = standard deviation.

**Table 2:** Factors associated with successful outcome after adhesiolysis using a steerable catheter

Variable	Univariable analysis		Multivariable analysis	
	OR (95% CI)	*p* value	Adjusted OR (95% CI)	*p* value
Duration of pain (months)	0.997 (0.992-1.002)	0.223		
Diabetes	1.076 (0.515-2.250)	0.845		
Spondylolisthesis	0.458 (0.193-1.085)	0.076	1.929 (0.400-9.311)	0.413
Central stenosis				
Grade				
Mild	1 (*Ref*)			
Moderate	1.000 (0.382-2.616)	1.000		
Severe	0.643 (0.238-1.735)	0.383		
**Foraminal stenosis**				
**Grade**				
Mild	1 (*Ref*)		1 (*Ref*)	
Moderate	0.530 (0.219-1.282)	0.159	0.441 (0.120-1.621)	0.217
Severe	0.310 (0.086-1.109)	0.072	0.137 (0.018-1.021)	0.052
Response to previous caudal block	3.039 (1.127-8.198)	0.028*	4.403 (1.330-14.579)	0.015*

* indicates significant difference; OR = odds ratio; CI = confidence interval; Ref = reference.

## Abbreviations

ASIPP: American Society of Interventional Pain Physicians
FBSS: Failed back surgery syndrome
MRI: Magnetic resonance imaging
NRS: Numerical rating scale
OR: Odds ratio
CI: Confidence interval
